# Synergistic anticancer activity of resveratrol-loaded polymeric nanoparticles and sunitinib in colorectal cancer treatment

**DOI:** 10.1098/rsos.241817

**Published:** 2025-04-23

**Authors:** Punnida Nonsuwan, Nattarika Niwetbowornchai, Kanyanut Insawang, Natsuda Kunwong, Kornrawee Srichan, Chatchawan Srisawat, Paweena Dana, Nattika Saengkrit, Kytai T. Nguyen, Primana Punnakitikashem

**Affiliations:** ^1^ Department of Biochemistry, Mahidol University Faculty of Medicine Siriraj Hospital, Bangkok 10700, Thailand; ^2^ Siriraj Center of Research Excellence in Theranostic Nanomedicine, Faculty of Medicine Siriraj Hospital, Mahidol University, Bangkok 10700, Thailand; ^3^ National Nanotechnology Center (NANOTEC), National Science and Technology Development Agency (NSTDA), Pathumthani 12120, Thailand; ^4^ Department of Bioengineering, University of Texas at Arlington, Arlington, TX 76019, USA

**Keywords:** polymeric nanoparticles, drug combination, resveratrol, sunitinib, colorectal cancer

## Abstract

The development of novel and effective treatment strategies, particularly through drug combinations, can significantly enhance therapeutic outcomes. This study explores the innovative combination of resveratrol (RES), a phenolic compound, with sunitinib (SUNI), a multitarget tyrosine kinase inhibitor, for targeting human colon adenocarcinoma cell line HT-29. We identified a synergistic effect at a SUNI:RES ratio of 1:8, based on their half-maximal inhibitory concentration values. Increasing the dosage of the combined treatment led to a notable reduction in cell viability, observed in both two-dimensional (2D) and three-dimensional cell cultures. To improve RES therapeutic efficacy, drug-loaded polymeric nanoparticles (PLGA-RES) were successfully fabricated with an average diameter of 178.4 ± 4.6 nm. The combination of PLGA-RES and free SUNI at the optimal ratio exhibited enhanced anticancer activity, reducing cell viability by approximately 25 and 15% more than PLGA-RES and free SUNI alone, respectively, in 2D cultures. Moreover, this combination therapy demonstrated superior effectiveness in treating HT-29 spheroids over 24 and 48 h. These findings highlight the potential of this combined approach to improve colorectal cancer treatment outcomes.

## 1. Introduction

Colorectal cancer (CRC) is the third most common cancer in the world and the fourth leading cause of death, which is often found in developed countries [1,2]. The 5-year survival rate in the USA is higher than 60%, but it drops below 40% in developing countries [3]. This is problematic. Solutions and treatments must be explored to reduce this rate. CRC is treated using diverse approaches, such as surgery, radiation, chemotherapy and various modalities, including immunotherapy and targeted therapy [4].

Chemotherapy resistance poses a significant challenge in cancer treatment, particularly in metastatic CRC patients undergoing multiple lines of chemotherapy [5,6]. Combining various drugs in cancer therapy addresses this resistance by reducing dose-limiting single-agent toxicity and enhancing treatment efficacy by leveraging the diverse functions of these drugs [5,7]. Sunitinib (SUNI), a multitarget inhibitor of receptor tyrosine kinases, received Food and Drug Administration (FDA) approval in 2006 for treating renal cell carcinoma and gastrointestinal stromal tumours [8]. Ongoing clinical trials are assessing its effectiveness against other tumour types, including metastatic colon cancer [9]. It was observed that SUNI, a small-molecule inhibitor targeting multiple tyrosine kinases, including vascular endothelial growth factor receptors and platelet-derived growth factor receptors, induced apoptosis and hindered the growth of colon cancer cells [10]. To gain high therapeutic efficiency and reduce the chemoresistance in CRC, there is a need to identify novel therapeutic agents that can potentially be used in combination with SUNI during CRC treatment. This is crucial for enhancing patient survival and minimizing adverse effects.

Recently, herbal extracts have been used as alternative therapeutic agents in a number of diseases including cancer. Resveratrol (RES), a natural polyphenol flavonoid present in red grapes, red wine, berries, nuts and other sources, exhibits potent antioxidant, anti-inflammatory and anti-tumour properties [11]. Its anti-fibrotic effects have been demonstrated in various organs, including the lungs, liver and kidneys, according to multiple sources [12–16]. Furthermore, RES has been shown to inhibit the myofibroblast phenotype in primary human fibrotic buccal mucosal fibroblasts [17]. In the context of cholangiocarcinoma, RES has been reported to affect the tumour microenvironment (TME) by inhibiting interleukin-6 secretion and disrupting cross-talk interactions [18]. RES has demonstrated effectiveness as a chemo-preventive agent for CRC [19,20]. It hinders the formation of carcinogen-induced preneoplastic lesions known as aberrant crypt foci in the mouse colon and suppresses the development of intestinal tumours (adenomas) in mice [19,21,22]. In addition, the growth inhibitory effects of RES are believed to be primarily from the induction of apoptosis [23,24]. To increase the anticancer activity, the combination of RES with other drugs had been reported, such as cisplatin, doxorubicin, docetaxel and paclitaxel [25–29]. In this study, the combination of SUNI and RES for synergistic efficacy in CRC therapy s reported for the first time.

Despite being used in clinical trials for various cancers, including CRC, the effectiveness of RES remains unsatisfactory [30]. This limitation may be attributed to its rapid metabolism, leading to a short half-life in blood plasma (8 min to 1.5 h), as well as its low water solubility, resulting in low bioavailability [31–33]. Therefore, strategies to enhance the pharmacokinetic profile of RES are required to overcome these obstacles and achieve effective anti-tumour activity. Recently, nanocarriers have gained rapid recognition for their ability to enhance drug bioavailability and solubility, as well as to improve drug stability, particularly for highly hydrophobic drugs [34]. Various versatile delivery systems such as liposomes, micelles, and polymeric and inorganic nanoparticles (NPs) have been devised and investigated [35–38]. Among these, poly(D,L-lactic-*co*-glycolic acid) (PLGA), an FDA-approved biocompatible polymer, has been extensively studied as a drug carrier [39,40]. PLGA NPs are a preferred choice for developing a rational cancer theranostic system due to their favourable characteristics such as biocompatibility, passive targeting, surface modifiability and controlled drug release kinetics [40,41].

The main goal of this study was to investigate the synergistic efficacy of the therapeutic agent RES and anticancer drug SUNI. We developed and characterized RES-loaded polymeric NPs (PLGA-RES) as well as characterizing their particle size, zeta potential, *in vitro* drug release profile, encapsulation efficiency, cellular uptake and biocompatibility. Furthermore, we evaluated the *in vitro* anticancer activities in two-dimensional (2D) and three-dimensional (3D) cultures against human colon adenocarcinoma (HT-29) cell line. The inhibitory effect of the combination of PLGA-RES and SUNI on cancer cell proliferation suggests that the synergism of RES and SUNI has promising pharmacological potential for treating CRC.

## 2. Material and methods

### 2.1. Materials

PLGA (molar ratio of D,L-lactic to glycolic acid, 50:50; molecular weight, 24–38 kDa), poly(vinyl alcohol) (PVA; average molecular weight of 31−50 kDa) and coumarin-6 (Cou6) were purchased from Sigma-Aldrich (St Louis, MO, USA). RES and McCoy’s 5A medium were acquired from Merck (Darmstadt, Germany). Sunitinib malate (SUNI) was obtained from MedChemExpress (Monmouth Junction, NJ, USA). Fetal bovine serum (FBS) was bought from Cytiva (Marlborough, MA, USA). Penicillin–streptomycin was obtained from Gibco (Grand Island, NY, USA). CellTiter-Blue^®^ cell viability assay and CellTiter-Glo assay were purchased from Promega (Madison, WI, USA).

### 2.2. Evaluation of drug combination effects

HT-29 cells were suspended in 200 µl McCoy’s 5A medium at a concentration of 1 × 10^4^ cells per well in 96-well culture plates and allowed to attach for 24 h at 37°C, 5% CO_2_, in an incubator. For single-drug treatment, SUNI (0.5−64 µM) and RES (4−512 µM) were added and incubated for 24 h. Cell viability was determined using CellTiter-Blue^®^ cell viability assays according to the manufacturer’s protocol. Culture medium was removed, fresh medium containing CellTiter-Blue reagents was added and then incubated at 37°C with 5% CO_2_ for 2 h. Fluorescence intensity was measured (545_Ex_/590_Em_) using a microplate reader (BioTek Synergy HT, USA).

To explore the synergistic efficacy of RES and SUNI against HT-29 cells, drugs were added at increasing concentrations while maintaining fixed ratios of their half-maximal inhibitory concentration (IC_50_) values. The combination index (CI) was determined based on the formula


$$CI=\;\frac{D_{1}}{D_{1x}}+\frac{D_{2}}{D_{2x}},$$

where D_1x_ and D_2x_ indicate the combination doses of drug 1 and drug 2 required to achieve *x*% inhibition as drug 1 (D_1_) and drug 2 (D_2_) when present alone. A CI value of <1, =1 and >1 indicates synergism, additive effect and antagonism in the combined drug action, respectively [42].

### 2.3. Treatment of HT-29 spheroids with sunitinib and resveratrol combination

Spheroids were generated from an initial suspension of 3000 cells per well. After a 3-day incubation period, combination treatments of SUNI and RES were applied at a SUNI:RES ratio of 1:8. SUNI was introduced at three concentrations (4, 8 and 16 µM), while RES was applied at 32, 64 and 128 µM. The changes in spheroid diameter were evaluated by inverted light microscopy (Nikon Eclipse Ti-S, USA) at 24 and 48 h after treatment. The diameters of the spheroid edges were measured using ImageJ software.

### 2.4. Preparation of resveratrol-loaded poly(D,L-lactic-*co*-glycolic acid) nanoparticles

RES-loaded PLGA nanoparticles (PLGA-RES) were synthesized using the solvent evaporation technique. Briefly, PLGA (5% w/v) was dissolved in dichloromethane, and RES solution (0.4% w/v) was added. This organic phase was then added dropwise under stirring to an aqueous phase consisting of 2% (w/v) PVA and sonicated at 20% amplitude for 10 min. The emulsion was left for 4 h to evaporate the organic phase. The NPs were collected by centrifugation at 12 000 r.p.m. for 20 min, resuspended with a small amount of MilliQ water and then lyophilized. The freeze-dried products were stored at −20°C for further use. The PLGA NPs without drugs were prepared following the same protocol.

### 2.5. Characterization of nanoparticles

The lyophilized NPs were dispersed in MilliQ water, and the concentration was 1 mg ml^−1^. Particle size, polydispersity index (PDI) and zeta potential of NPs were investigated by a Zetasizer Nano-ZS90 (Malvern Instruments, UK) using disposable zeta sizer cuvettes. To determine the stability of NPs, they were kept at 4°C for three months and the particle size, PDI and zeta potential were analysed as mentioned above.

The morphology of PLGA NPs and PLGA-RES was characterized by transmission electron microscopy (TEM; Hitachi/HT7800, Japan). The NPs were diluted to a concentration of 1 mg ml^−1^ and placed onto Formvar-coated copper grids followed by staining with uranyl acetate. The NPs were dried overnight and observed under TEM.

To evaluate the percentage encapsulation efficiency (%EE) of PLGA-RES, 1 mg of NPs was dissolved in dimethyl sulfoxide (DMSO) and probe sonicated with 30% amplitude for 1 min to destroy the NPs’ structure, and encapsulated RES was completely released. The sample was centrifuged at 12 000 r.p.m. for 15 min. The supernatant was then collected, and the absorbance of the solution was read at 320 nm. The content of the drug was calculated from the standard curve of RES solution at various concentrations dissolved in DMSO. The %EE was determined by the following equation:



$$\%EE=(W_{E}/W_{T})\times 100,$$

where *W*_*E*_ and *W*_*T*_ are the amount of RES encapsulated in PLGA-RES NPs and the total amount of RES used for NP preparation, respectively.

The *in vitro* release was studied in 1× PBS. Briefly, 1 ml of NPs was placed into dialysis bags (molecular weight cutoff of 20 kDa; Repligen). Then the dialysis bags were put into 20 ml release medium and maintained in an orbital shaker bath at 37°C and 100 r.p.m. At different time points, 1 ml aliquots of the release solution were collected and replaced by 1 ml fresh release medium. The absorbance was measured at 320 nm. The amount of RES in the release medium was calculated by comparing to the RES standard curve, and the cumulative release was determined.

### 2.6. *In vitro* cytotoxicity assay of nanoparticles

HT-29 cells were cultured in McCoy’s 5A medium supplemented with 10% FBS and 1% penicillin–streptomycin. The cells were incubated in a humidified atmosphere of 5% CO_2_ at 37°C. The cytotoxicity of PLGA NPs, PLGA-RES, combination of PLGA-RES and SUNI (PLGA-RES + SUNI) and free drugs on HT-29 was measured by CellTiter-Blue^®^ assay. HT-29 cells were seeded at 1 × 10⁴ cells per well in 96-well plates. After cell adherence, samples were added and incubated for 24 h before measuring cell viability.

### 2.7. Cellular uptake

To study the cellular uptake efficiency of prepared NPs in HT-29 cells, inverted light microscopy was carried out. PLGA NPs loaded with Cou6 (PLGA-Cou6) were formulated using the same protocol as described in §2.4. The HT-29 cells were seeded into 24-well plates (5 × 10^4^ cells per well) and incubated overnight to allow cell attachment. Different concentrations of Cou6-loaded formulation were incubated with cells for 4 h followed by washing with PBS. The cells were then fixed with 4% paraformaldehyde for 10 min and stained with nucleus-staining dye, DAPI, for 15 min. The stained well plate was then observed for fluorescence intensity at ×20 magnification.

### 2.8. Nanoparticle treatment on HT-29 spheroids

HT-29 cells (3000 cells per well) were seeded into 96-well round-bottom ultra-low attachment plates and incubated at 37°C, 5% CO_₂_ for 3 days to form spheroids. Spheroids were treated with PLGA NPs, PLGA-RES, PLGA-RES + SUNI and free drugs for 24 and 48 h. Treated spheroid sizes were observed by inverted microscopy and measured using ImageJ software.

### 2.9. Statistical analysis

The mean ± standard deviation (s.d.) with one-way ANOVA was performed to analyse the statistical differences between data. Data are presented as the mean ± s.d. (*n* = 6). Significance was set at *p < 0.05, **p < 0.01, and ****p < 0.0001.

## 3. Results and discussion

### 3.1. Synergy of drugs *in vitro*

**Figure 1.**
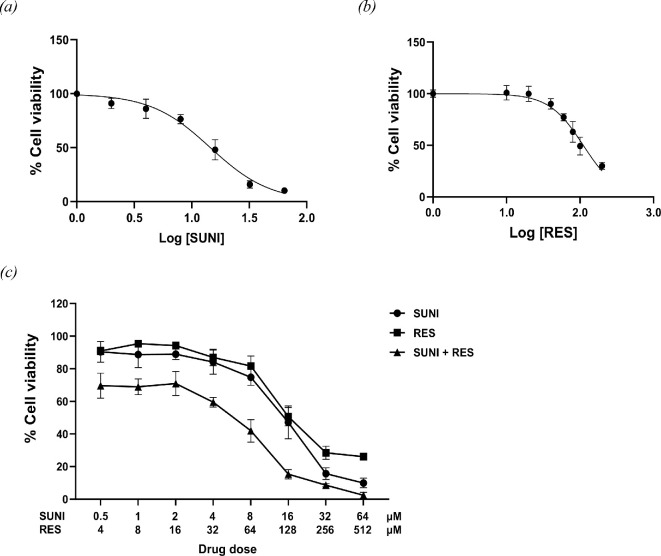
Dose-dependent cytotoxicity. (*a*) SUNI and (*b*) RES treatment at 24 h. HT-29 cells were treated with various concentrations of SUNI and RES. (*c*) Effect of combination treatment with SUNI and RES. A fixed concentration ratio of SUNI:RES at different concentrations were used for treatment for 24 h.

The effects of SUNI and RES on the viability of HT-29 cells were determined by treatment with increasing concentrations of SUNI (0.5−64 µM) and RES (4−512 µM) for 24 h. As shown in figure 1*a,b*, the viability of HT-29 cells was inhibited by SUNI and RES in a dose-dependent manner. The presence of a high dose caused a higher reduction of cell viability than as a single agent. Therefore, to improve the therapeutic effect over single-drug treatment, the combination of SUNI and RES for inhibiting the growth of HT-29 cells was studied.

To evaluate the effect of combination drugs on the survival of HT-29 cells, the IC_50_ of SUNI and RES was calculated to be a fixed ratio of combination. Due to the IC_50_ of SUNI (log IC_50_ = 1.158) and RES (log IC_50_ = 2.045) being 14.40 and 110.9 μM, respectively, the concentration ratio of SUNI:RES was 1:8. By fixing the ratio of the IC_50_ value (1:8), different concentrations of RES and SUNI were combined and used for treatment of HT-29 cells for 24 h. Figure 1*c* shows that treatment of HT-29 cells with SUNI plus RES resulted in a decrease in cell viability in all concentration ratios, which was greater than either SUNI or RES alone. The CI confirmed the synergy observed with SUNI and RES as shown in table 1. CI of 1.0 reflects additive effects, whereas values greater than and less than 1.0 indicate antagonism and synergism, respectively. The results indicated the synergistic (CI < 1) effect of SUNI combined with RES, with CI values ranging from 0.11 to 0.82 at different dose combination ratios. Our results align with earlier studies, indicating a synergistic inhibitory impact on the proliferation of colorectal cells when combining RES with other anticancer agents like 5-fluorouracil (5-FU), cetuximab and didox, as observed in previous reports [43–46]. RES seem to act as a chemosensitizer for CRC cells to chemotherapeutic agents and therefore enhances the therapeutic effect. Moreover, to investigate the treatment within a simulated TME, the impact of SUNI, RES and their combination on a 3D spheroid model was evaluated.

**Table 1.** Evaluation of drug interactions of SUNI and RES in HT-29 cell line.

SUNI (µM)	0.5	1	2	4	8	16	32	64
RES (µM)	4	8	16	32	64	128	256	512
total dose (µM)	4.5	9.0	18.0	36.0	72.0	144.0	288.0	576.0
Fa	0.142	0.187	0.213	0.469	0.495	0.842	0.904	0.998
CI value	0.216	0.327	0.571	0.445	0.821	0.478	0.647	0.108
interpretation	strong synergism	synergism	synergism	synergism	moderate synergism	synergism	synergism	strong synergism

CI values are generated over a range of Fa levels from 0.05 to 0.90 (5–90% growth inhibition).

CI of 1 indicates an additive effect between two agents.

CI < 1 indicates synergism.

CI > 1 indicates antagonism.

### 3.2. Effects of combination therapy on HT-29 spheroids

**Figure 2.**
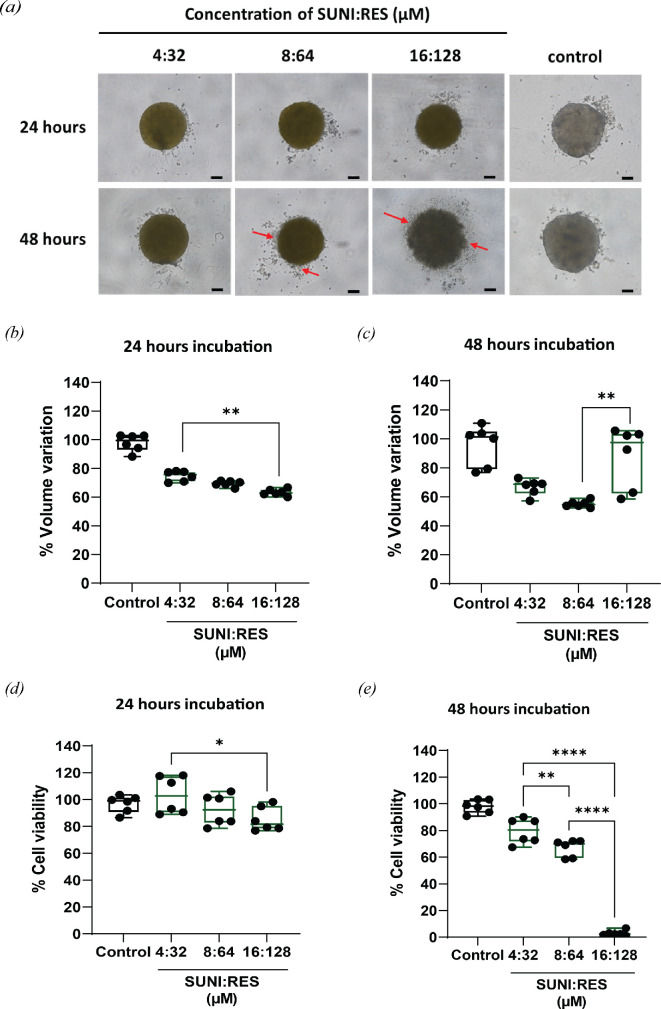
The effects of combined drugs on spheroids. (*a*) Brightfield images for 24 and 48 h incubation. Scale bar, 100 μm. The volume of HT-29 cell spheroids for (*b*) 24 and (*c*) 48 h incubation. Cytotoxicity evaluation of HT−29 cell spheroids for (*d*) 24 and (*e*) 48 h incubation (*p < 0.05, **p < 0.01 and ****p < 0.0001).

Three different concentrations of SUNI + RES of 4 + 32, 8 + 64 and 16 + 128 µM were selected to study the effect on 3D spheroids because the viability of cells was clearly seen in a dose-dependent manner in this range (figure 1*c*). The spheroid morphology after treatment with the combination of SUNI and RES was monitored by inverted light microscopy (figure 2*a*). The diameters of the spheroid edges were measured initially as pixels using ImageJ software and converted to micrometres by comparison to a reference length. The smaller the size of the spheroid, the lower the proliferation of cells [47]. The volume of spheroids was calculated and reported. The growth of the spheroids is presented in figure 2*b,c*. The study demonstrated that a more significant decline in cell viability was observed with increased concentrations of SUNI + RES. Moreover, the higher dosage of treatment has the potential to destabilize the spheroids, particularly with extended drug exposure and incubation periods. The disruption of the spheroid’s smooth surface integrity was due to weaker attachment and direct exposure to drug treatments [48]. The outer dead-cell layers of the spheroid likely started to detach (indicated with red arrows) due to drug-mediated cytotoxicity, resulting in a bigger size. This was clearly seen in the treatment of SUNI plus RES at higher concentrations for 48 h of treatment time. Besides decreasing in size of spheroids, the cell viability in figure 2*d,e* confirmed that the higher the dose of combined treatment, the greater the decrease in cell viability of HT-29 cells.

### 3.3. Preparation and characterization of nanoparticles

**Figure 3.**
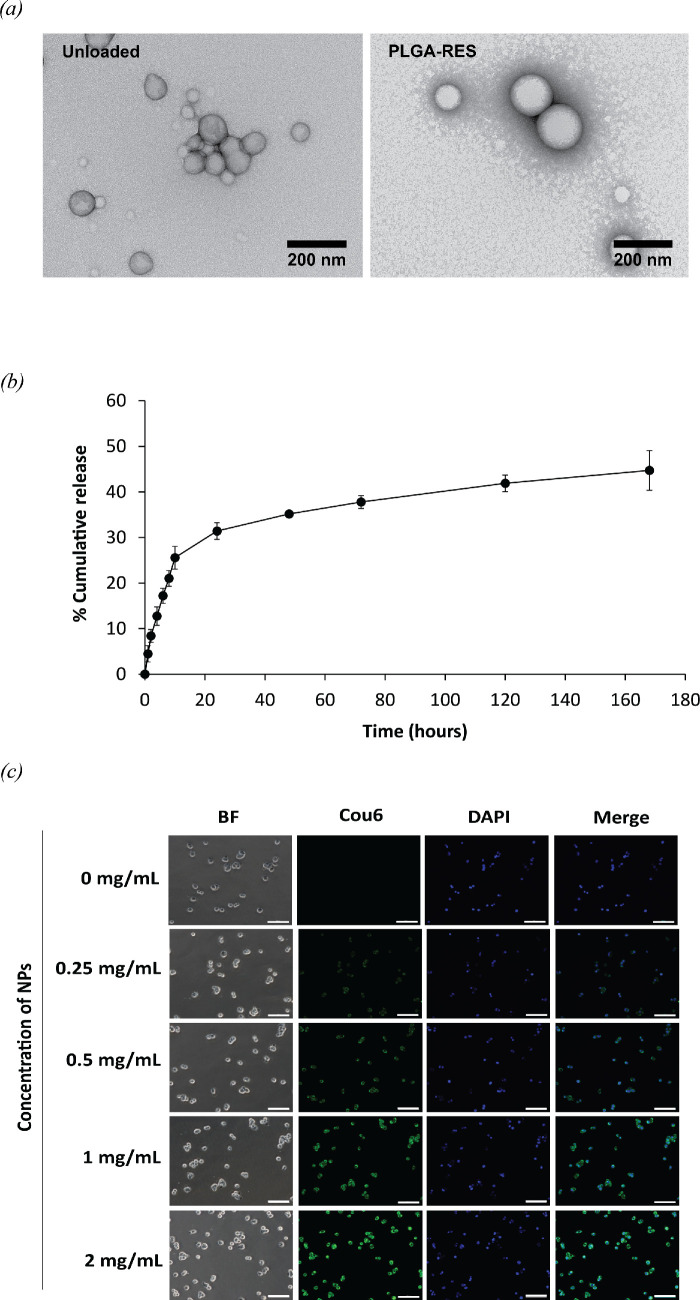
Characterization of NPs. (*a*) TEM images of unloaded NPs (PLGA; left) and RES-loaded NPs (PLGA-RES; right) at ×50 000 magnification. (*b*) *In vitro* release of RES from PLGA NPs. (*c*) Fluorescence microscopy uptake images of Cou6-loaded PLGA NPs in HT-29 cells at 4 h (scale bar, 100 µm).

The facile emulsified solvent evaporation method was used to prepare NPs in this study. Due to biocompatibility, low toxicity and biodegradability of PLGA, it has been widely used as a carrier in drug delivery systems. To enhance the stability of the drug, solubility and treatment efficacy, RES was encapsulated in PLGA. The PLGA-RES NPs were monodispersed with an average particle size of 178.4 ± 4.6 nm and a PDI of 0.081 ± 0.004 and that of unloaded NPs was 182.0 ± 2.2 nm with a PDI of 0.067 ± 0.035 (table 2). Both types of NPs were spherical (figure 3*a*) with a negatively charged surface of −26.6 ± 0.9 and −24.0 ± 3.9 mV for PLGA-RES and PLGA NPs, respectively (table 2). The negative charge observed can be attributed to the presence of free carboxyl groups from the surface of PLGA NPs [49,50]. The higher zeta potential measurement of the particles indicates higher level of stability. Compared to SUNI that has a half-life of 40−70 h [51,52], the short biological half-life of RES (8 min to 1.5 h) was reported [33,53]. It was rapid metabolism and elimination from the blood pool and low aqueous solubility resulting in limiting its bioavailability and efficiency against cancer cells [53,54]. Therefore, the polymeric NPs were designed to encapsulate RES in order to enhance its biological half-life and systemic circulation time. In this study, the synergistic effect of the combination of RES-loaded PLGA NPs and free SUNI against CRC cell lines was investigated as detailed in §3.4.

**Table 2.** Hydrodynamic diameter, zeta potential and PDI of PLGA and PLGA-RES.

formulated NPs	time (month)	average size (nm)	PDI	zeta potential (mV)
PLGA	0	182.0 ± 2.2	0.067 ± 0.035	−24.0 ± 3.9
	3	181.4 ± 0.6	0.046 ± 0.037	−22.5 ± 1.5
PLGA-RES	0	178.4 ± 4.6	0.081 ± 0.004	−26.6 ± 0.9
	3	178.0 ± 8.6	0.080 ± 0.087	−21.1 ± 1.6

The amount of encapsulated RES was calculated by dissolving the PLGA-RES in DMSO, and the values obtained by the spectrophotometric analysis were calculated with the calibration line equation. The NPs carried an amount of 0.018 mg of RES per mg of PLGA with %EE of 22.76 ± 1.69%. The outcome corresponded to that of an earlier report, which indicated around 24% EE when employing the identical RES:PLGA ratio [55]. However, the %EE for various hydrophobic drugs, including curcumin, paclitaxel and doxorubicin, in PLGA exceeded 45% [56–58]. RES-loaded PLGA with %EE of 30–96% have been reported, which was higher than our observation [55,59]. The difference in %EE may be attributed to the drug:PLGA ratio and the structural characteristics of the drugs [55,58,60].

PLGA NPs and PLGA-RES showed insignificant differences in the parameters of particle size, PDI and zeta potential during 3 months of storage at 4°C (table 2). This indicates that the formulated NPs were highly stable for 3 months. Our findings align with the conclusions of a prior study, indicating that PLGA-RES maintains colloidal stability for a duration ranging from 7 days to 2 months [55,61].

The *in vitro* drug release behaviour of PLGA-RES is illustrated in figure 3*b*. The sustained release pattern without any burst release of PLGA-RES was obtained. The cumulative percentage of drug release after 48 h was measured at only 35.15 ± 0.25% and sustained released to 44.72 ± 4.37% over 168 h. The absence of an initial burst release is likely attributed to there being fewer RES molecules present on the surface of NPs. The overall slow-release profile in this study seems to have a similar pattern to previous reports highlighting a sustained and slow-release process [53,55].

Subsequently, in order to comprehend the process behind RES release from the PLGA NPs, different kinetic models were investigated. The *in vitro* release data were analysed using the zero-order, first-order, Higuchi, Korsmeyer–Peppas and Weibull models (table 3) [62–64]. Among these models, the Weibull model emerged as the most suitable for fitting our data, displaying the highest correlation coefficient (*R*²) and the lowest sum-of-square residuals (SSR). In addition, the parameter *β* is useful for the determination of the drug release mechanism. The limits considered were *β* ≤ 0.56, which correspond to the mechanisms of Fickian diffusion [62,65]. Our results agree with a previous report demonstrating the ability of the Weibull model to describe the release patterns of PLGA-based matrices [62].

**Table 3.** Kinetic data analysis of RES release from NPs.

particle	zero-order model (*Q*_*t*_ = *K*_0_*t* + *Q*_0_)			first-order model (ln *Q*_*t*_ = −*K*_1_*t* + ln *Q*_0_)			Higuchi model (*Q*_*t*_ = *K*_*H*_*t*^1/2^ + *Q*_0_)			Korsmeyer–Peppas model (ln *Q*_*t*_ = *n* ln *t* + ln *K*)			Weibull model (ln(−ln(1 *− Q*_*t*_/100)) = *β* ln *t* + ln *α*)		
	*R* ^2^	*K*_0_	SSR	*R* ^2^	*K*_1_	SSR	*R* ^2^	*K*_*H*_	SSR	*R* ^2^	*n*	SSR	*R* ^2^	*β*	SSR
PLGA-RES	0.707	0.207	558.7	0.472	0.009	2.844	0.871	3.154	244.8	0.896	0.409	0.560	0.917	0.462	0.555

Cou6-loaded PLGA NPs (PLGA-Cou6) were prepared for the cell internalization study. Cellular uptake images of PLGA-Cou6 (green) were visualized by the fluorescein isothiocyanate channel, and the cell nucleus can be observed with the DAPI channel (blue) as shown in figure 3*c*. PLGA-Cou6 could be internalized into HT-29 cells after 4 h of incubation and found to be highly concentrated in cytoplasm, taking on a light green surrounding the nucleus upon overlay. The results show that the uptake was significantly increased with an increasing PLGA-Cou6 concentration. The results demonstrated that PLGA NPs had a good cellular internalization and delivery ability.

### 3.4. *In vitro* cytotoxicity assay of nanoparticles

**Figure 4.**
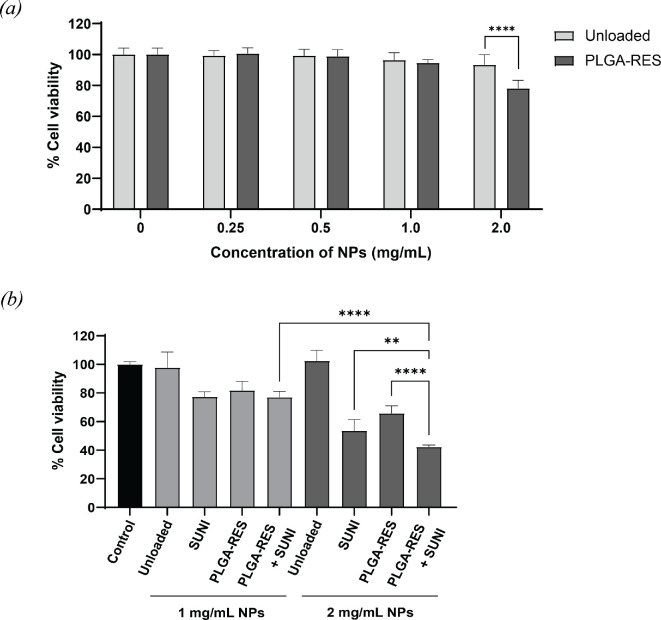
HT-29 cell viability after 24 h. (*a*) Effect of concentration of PLGA NPs and (*b*) cell cytotoxicity of the combination of RES-loaded PLGA NPs and free SUNI (**p < 0.01 and ****p < 0.0001).

CellTiter-Blue^®^ assays were conducted to determine cellular toxicity of the PLGA, PLGA-RES and RES drug solution against the HT-29 cell line. The unloaded PLGA NPs had no toxicity to cells in the concentration range of 0.25−2 mg ml^−1^ (figure 4*a*). This further demonstrated that the decreasing of cell viability depends on the RES concentration released from PLGA-RES in this concentration range of NPs. The concentration of NPs started to have an impact on cell viability at 1 mg ml^−1^ PLGA-RES and was obviously seen at 2 mg ml^−1^ (figure 4*a*).

In this work, the concentration of PLGA-RES at 1 and 2 mg ml^−1^ contained RES of 64 and 128 μM, respectively. The combination of RES-loaded PLGA NPs and free SUNI was further studied to see the synergistic effect on cytotoxicity. As described in §3.1, the concentration ratio of SUNI:RES to perform the synergy was 1:8. Therefore, PLGA-RES of 1 and 2 mg ml^−1^ was combined with free SUNI at a concentration of 8 and 16 μM, respectively. The effects of cytotoxicity induced by PLGA, PLGA-RES and the combination of PLGA-RES and free SUNI after treating the cells with different doses of NPs and drugs for 24 h were assessed, as shown in figure 4*b*. The observations showed that the combination of PLGA-RES and free SUNI-treated cells exhibited growth inhibitory effects in HT-29 cells, and these effects were more significantly pronounced when using higher doses of PLGA-RES (2 mg ml^−1^) and SUNI (16 μM). The cell viability of the combined treatment was around 25 and 15% lower than that of PLGA-RES (2 mg ml^−1^) and SUNI (16 μM), respectively. These results of synergy go in a similar direction to those of the free drug combination previously described in §3.1. However, the combination of NPs and free SUNI provided lower toxicity than the combination of free RES and SUNI at the same concentration (figure 1*c*). For example, the ratio of SUNI:RES at 16 μM:128 μM corresponded to 16 μM SUNI combined with 2 mg ml^−1^ of PLGA-RES, free drug combination showed 17% viability while particles plus free drug showed 40% viability. The reason behind this might be that the concentration of RES released from PLGA is less than the actual value due to its sustained release.

### 3.5. Effects of combination of resveratrol-loaded poly(D,L-lactic-*co*-glycolic acid) nanoparticles and free sunitinib on HT-29 spheroids

**Figure 5.**
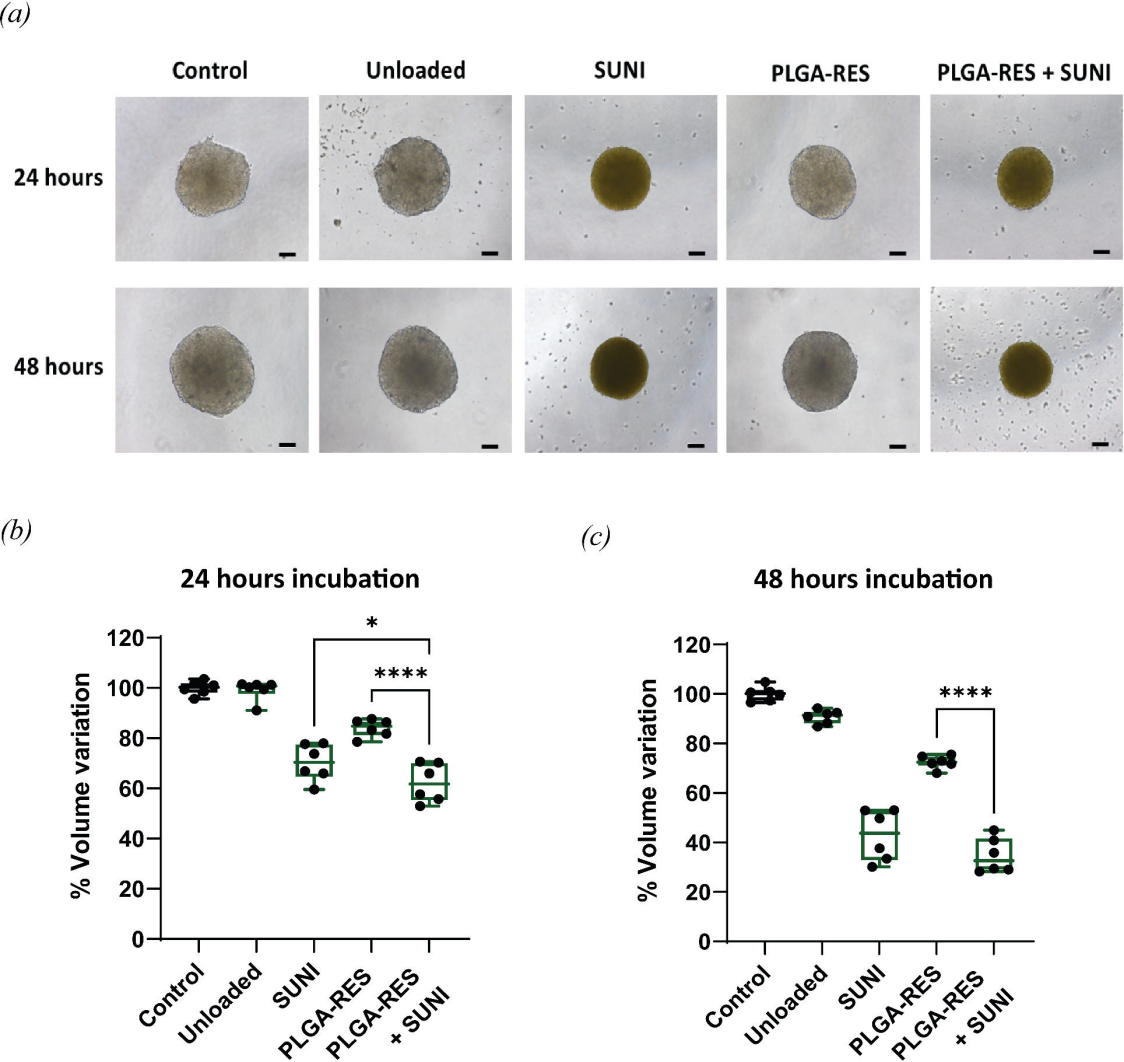
Effects of free drug and drug-loaded PLGA NPs on 3D spheroids after 24 and 48 h incubation. (*a*) Macroscopic images of spheroids observed by inverted microscopy. Scale bar, 100 µm. The volume of HT-29 cell spheroid for (*b*) 24 and (*c*) 48 h began with the same number of seeding cells (*p < 0.05 and ****p < 0.0001).

The effects of unloaded PLGA, PLGA-RES and the combination of PLGA-RES and SUNI on cell viability were investigated in 3D spheroids. In §3.4, the viability of HT-29 cells was significantly reduced at a concentration of 2 mg ml^−1^ NPs. Consequently, the dose of NPs at 2 mg ml^−1^ was used to treat HT-29 cell spheroids. As shown in figure 5, the HT-29 spheroids reduced in size after incubation with PLGA-RES and PLGA-RES plus free SUNI for 24 and 48 h. In comparison to unloaded PLGA, PLGA-RES treatment appears to have little impact on inhibiting spheroid growth, and spheroids treated with free SUNI exhibited noticeably smaller sizes. Notably, the combination of PLGA-RES and free SUNI demonstrated a heightened effect on HT-29 spheroids, resulting in the smallest spheroid size compared to the uncombined treatment. This synergistic effect contributes to the inhibition of spheroid growth. Our present findings are similar to the results of a previous report, suggesting that the combination of RES-loaded liposome and 5-FU enhances sensitivity in treating CRC spheroids [66].

## 4. Conclusion

In conclusion, this comprehensive study emphasizes a nanoformulation approach to load a natural ingredient, RES, and combine it with a chemotherapy drug, SUNI, aiming for effective delivery and combined effectiveness against CRC cells. The combination ratio of SUNI:RES of 1:8 led to a synergism to reduce the viability of HT-29 cells. Moreover, the combination of RES-loaded PLGA and free SUNI demonstrated higher cytotoxicity against HT-29 cells compared to individual treatment in both 2D and 3D cell cultures. Hence, this simultaneous delivery system could offer a novel approach to treat CRC.

## Data Availability

Data are available at Dryad [67].
